# Assessing Specific Oligonucleotides and Small Molecule Antibiotics for the Ability to Inhibit the CRD-BP-CD44 RNA Interaction

**DOI:** 10.1371/journal.pone.0091585

**Published:** 2014-03-12

**Authors:** Dustin T. King, Mark Barnes, Dana Thomsen, Chow H. Lee

**Affiliations:** Chemistry Program, University of Northern British Columbia, Prince George, British Columbia, Canada; CNRS UMR7275, France

## Abstract

Studies on Coding Region Determinant-Binding Protein (CRD-BP) and its orthologs have confirmed their functional role in mRNA stability and localization. CRD-BP is present in extremely low levels in normal adult tissues, but it is over-expressed in many types of aggressive human cancers and in neonatal tissues. Although the exact role of CRD-BP in tumour progression is unclear, cumulative evidence suggests that its ability to physically associate with target mRNAs is an important criterion for its oncogenic role. CRD-BP has high affinity for the 3′UTR of the oncogenic CD44 mRNA and depletion of CRD-BP in cells led to destabilization of CD44 mRNA, decreased CD44 expression, reduced adhesion and disruption of invadopodia formation. Here, we further characterize the CRD-BP-CD44 RNA interaction and assess specific antisense oligonucleotides and small molecule antibiotics for their ability to inhibit the CRD-BP-CD44 RNA interaction. CRD-BP has a high affinity for binding to CD44 RNA nts 2862–3055 with a K_d_ of 645 nM. Out of ten antisense oligonucleotides spanning nts 2862–3055, only three antisense oligonucleotides (DD4, DD7 and DD10) were effective in competing with CRD-BP for binding to ^32^P-labeled CD44 RNA. The potency of DD4, DD7 and DD10 in inhibiting the CRD-BP-CD44 RNA interaction *in vitro* correlated with their ability to specifically reduce the steady-state level of CD44 mRNA in cells. The aminoglycoside antibiotics neomycin, paramomycin, kanamycin and streptomycin effectively inhibited the CRD-BP-CD44 RNA interaction *in vitro*. Assessing the potential inhibitory effect of aminoglycoside antibiotics including neomycin on the CRD-BP-CD44 mRNA interaction in cells proved difficult, likely due to their propensity to non-specifically bind nucleic acids. Our results have important implications for future studies in finding small molecules and nucleic acid-based inhibitors that interfere with protein-RNA interactions.

## Introduction

The coding region determinant-binding protein (CRD-BP), also commonly known as insulin-like growth factor II mRNA-binding protein 1 (IMP1), is a member of the conserved VICKZ family of RNA-binding proteins that are characterized by the presence of two N-terminal RNA-recognition motifs (RRMs) followed by four C-terminal KH [hnRNP (heterogenous nuclear ribonucleoprotein) K-homology] domains [1]–[4]. CRD-BP has about 95% protein sequence identity to the chicken zipcode-binding protein 1 (ZBP1) [2] that associates with β–actin mRNA [5], [6].

Mice deficient in IMP1 developed dwarfism and exhibited severe impairment in intestinal phenotype at early birth [7]. This is consistent with the observation that CRD-BP is abundantly expressed in fetal tissues but not in normal adult tissues [8]. The study also suggests that this RNA-binding protein plays a significant role in intestinal development of the fetus. Studies in *Drosophila melanogaster*, *Xenopus laevis* and mammalian neuronal cells have provided evidence for the role of IMP1 in nervous system development [1]. The ability of IMP1 to promote neurite outgrowth and branching is predominantly due to its role in controlling specific mRNA localization and translation, in particular the β–actin mRNA [1].

CRD-BP/IMP1 in human cancers has been extensively studied. While CRD-BP is virtually absent or undetectable in normal adult tissues, it is over-expressed in many types of human cancers including cancers of the breast [9], colon [10]–[12], brain [13], lung [13], [14], testicular [15], skin [16], ovarian [17], and chorion [18]. Furthermore, transgenic mice carrying targeted expression of CRD-BP develop mammary tumours [19], further implicating this RNA-binding protein in the development of cancer. Using colorectal cancer cell xenografts, it was recently demonstrated that IMP1 overexpression promotes xenograft tumour growth and dissemination into the blood [20].

The exact mechanism whereby CRD-BP regulates tumour growth and metastasis is still unclear although cumulative evidence suggests its role at the level of post-transcriptional regulation, particularly in its ability to stabilize oncogenic mRNAs. For instance, CRD-BP binds to the coding region of *βTrCP1* mRNA and over-expression of CRD-BP leads to stabilized *βTrCP1* mRNA, and elevated βTrCP1 protein levels, resulting in suppression of apoptosis in colorectal cancer cells [21]. Similarly, CRD-BP binds to and stabilizes *GLI*1 mRNA leading to elevated GLI1 protein, transcriptional activation, and proliferation of colorectal cancer cells [22]. CRD-BP is known to have high affinity for a specific coding region in c-*myc* mRNA [23], [24]. In breast cancer cells down-regulation of CRD-BP resulted in decreased c-Myc expression and reduced cell proliferation rates [25]. CRD-BP has been shown to bind to the coding region and 3′ untranslated region (UTR) of *K-Ras* mRNA and its overexpression led to increases in c-Myc and K-Ras expression as well as colon cancer cell proliferation [12]. The 3′UTR of MITF mRNA is also a binding site for CRD-BP and this interaction is critical for protecting the MITF transcript from degradation by miR-340, a mechanism believed to be important in melanocytes and malignant melanoma [26]. CRD-BP also has high affinity for a coding region of the *MDR*1 mRNA [24]. Consistent with the model that CRD-BP protects *MDR*1 mRNA from degradation is the observation of sensitization of drug-resistant cells to chemotherapeutic drugs upon down-regulation of IMP1 and MDR1 expression [27]. Lastly, CRD-BP binds with high affinity to several sites in the 3′UTR of CD44 mRNA, and depletion of CRD-BP leads to unstable CD44 mRNA, decreased CD44 protein, reduced cellular adhesion and invadopodia [28]. This is consistent with recent reports supporting the role of CRD-BP as a possible mediator of cancer cell metastasis and invasion. In addition to promoting colorectal cancer cell dissemination into the blood [20], knockdown of IMP1 leads to inhibition of migration and invasion of choriocarcinoma cells [18]. Furthermore, inhibition of CRD-BP expression impaired the ability of metastatic melanoma cells to proliferate and invade in response to hypoxia [29].

On the contrary to its oncogenic role, there is also evidence of a potential tumour suppressive role for CRD-BP under certain circumstances. The loss of CRD-BP has been shown to lead to the induction of leukemia cell proliferation [30]. In human breast cancer cells, repression of ZBP1 leads to increased cell proliferation and migration [31], [32]. CRD-BP also binds to a group of mRNA transcripts involved in mitosis and cell growth [32]. ZBP1 was shown to bind to and stabilize β-catenin, P16-ARC and E-cadherin mRNAs in metastatic breast cancer cells, suggesting a role of CRD-BP in strengthening cell-cell contacts and thus inhibiting tumour-cell invasion [31]. All of these contradictory findings in the oncogenic and tumour suppressor role of CRD-BP suggest that the net effect of CRD-BP binding to RNA targets can be complex and is likely dependent on the abundance of selective mRNA targets that can differ between cell types. Thus, controlling CRD-BP expression alone may not bring about the desired phenotype in different cells. This observation illustrates the need to design therapeutic strategies to inhibit the specific function of CRD-BP by controlling its binding to selective RNA targets. To date, there are limited reports characterizing compounds that can break CRD-BP-RNA interactions. This is unfortunate because CRD-BP poses an excellent potential anti-cancer drug target, and breaking specific CRD-BP-RNA interactions could represent a novel therapeutic approach. In this study, we further characterize the CRD-BP-CD44 RNA interaction. We then test a panel of antisense oligonucleotides and small molecule antibiotics for the ability to interfere with CRD-BP binding at a specific high affinity region in the 3′UTR of CD44 mRNA. We show that particular antisense oligonucleotides that interfere with the CRD-BP-CD44 RNA interaction can indeed specifically suppress CD44 mRNA expression in cells.

## Materials and Methods

### Cell line

HeLa cervical cancer cells purchased from the American Type Culture Collection (Rockville, Maryland) were maintained in MEM (Life Technologies, Burlington, Ontario) supplemented with 10% fetal bovine serum in a humidified incubator at 37°C supplied with 5% carbon dioxide. Cells were routinely maintained in 25 cm^2^ or 75 cm^2^ tissue culture flasks and harvested by 0.25% trypsin/0.02% EDTA treatment when they were in logarithmic phase of growth for various experiments.

### Antisense oligonucleotides and primers

Table 1 shows sequences of the antisense oligonucleotides (DD1 to DD10) used in this study. Oligonucleotides used for the electrophoretic mobility shift assays were standard phosphodiester DNA derivatives. For work in cells, the oligonucleotides were synthesized as 2′-*O*-methyl derivatives with a phosphodiester backbone. Table 1 also shows the sequences of primers used to amplify the DNA template for use in synthesizing CD44 RNA Fragments 4, 5, 8, 9, and truncated Fragment 4 CD44 RNAs, as well as oligonucleotides P1 to P6. Oligonucleotides and primers were synthesized by Integrated DNA Technologies (IDT) Inc. (Coralville, Iowa).

**Table 1:** Sequences of oligonucleotides and primers.

Name	Sequences and location at 3′UTR of human CD44 mRNA
DD1	ATTATTAATTGGGCCCTAATTT (2862–2884)
DD2	ACGATCAAATTCTTGCTGATTA (2881–2902)
DD3	GGCCTCCAAGTGGGAACTGGAA (2902–2923)
DD4	TAGCACACCCGAGGGATGAAAGG (2921–2943)
DD5	TTTTGTTAGAAGCCATCCATAG (2942–2963)
DD6	GAATACATATGTGTAGTTTTTG (2959–2980)
DD7	GGAAAGGTTGGCGATCAGGAAT (2976–2997)
DD8	GTCCTTAGCTGGTGGGGGAAAG (2993–3014)
DD9	AGGCCCTATTAACCCTGGGAAAT (3015–3037)
DD10	AAATTTCCTCCCAGGGACCAGG (3034–3055)
F4_forward	GGATCCTAATACGACTCACTATAGGAAATTAGGGCCCAATTAA (2862–2880)
F4_reverse	AAATTTCCTCCCAGGGAC (3037–3055)
F5_forward	GGATCCTAATACGACTCACTATAGGGATGAGTTAAGTGCCTGG (3565–3583)
F5_reverse	CACATAAGTTTAGGTAAC (3817–3835)
F8_forward	GGATCCTAATACGACTCACTATAGGGATGAGTTAAGTGCCTGG (3982–3946)
F8_reverse	CACATAAGTTTAGGTAAC (4218–4236)
F9_forward	GGATCCTAATACGACTCACTATAGGACCAGATCCCGGAGTTGG (4646–4664)
F9_reverse	CGCACAAGAGTTCCGTAG (4892–4910)
P1_reverse	ACCAGGCCCTATTAACCC (2878–2896)
P2_reverse	TTAGCTGGTGGGGGAAAG (2903–2921)
P3_reverse	AGGAATACATATGTGTAG (2920–2938)
P4_reverse	GCCATCCATAGCACACCC (2962–2980)
P5_reverse	GCCTCCAAGTGGGAACTG (2991–3009)
P6_reverse	ACGATCAAATTCTTGCTG (3018–3036)

T7 RNA promoter sequences are underlined.

### Transfection of cells with antisense oligonucleotides

Transient transfection of oligonucleotides was carried out using Lipofectamine 2000 reagent (Life Technologies) as according to the manufacturer’s instructions. As a negative control, the Scrambled Negative (SN) oligonucleotide (IDT Inc.) was used in addition to DD1 and DD10 oligonucleotides, which had no or little effect on the CRD-BP-CD44 RNA interaction *in vitro*. For experiments to determine mRNA expression using quantitative real-time PCR, cells were plated onto 6-well plates in a total volume of 2 mL at a density of 5×10^4^ cells/mL. After 24 hours, the cell media were replaced with fresh MEM containing 10% fetal bovine serum and Lipofectamine/oligonucleotide mixture. A similar transfection protocol was applied to the cells after a further 24-hour period of incubation. Forty-eight hours after the first transfection, the total RNA extracted from cells was subjected to quantitative real-time PCR. For Western blot experiments, cells were seeded onto 100 mm dishes in a total volume of 10 mL at a density of 1×10^4^ cells/mL. Cells were transfected at 24 and 48 hours after plating with the oligonucleotides. For RNA immuno-precipitation experiments, the cells were plated onto 100 mm dishes in a total volume of 10 mL at a density of 50×10^4^ cells/mL. In some experiments, cells were then transfected with 10 μg of pcDNA-FLAG or pcDNA-FLAG-CRD-BP 24 hours after plating. Cells were then transfected with oligonucleotides 48 hours after plating. Seventy-two hours after plating, the cells were lysed and subjected to RNA immuno-precipitation as described below. The pcDNA-FLAG-CRD-BP plasmid containing the entire coding region plus parts of 3′untranslated region of mouse CRD-BP was a gift from Dr. Jeffrey Ross, University of Wisconsin.

### Purification of recombinant CRD-BP

The plasmid pET28b(+)-CRD-BP which contains the entire coding region of mouse CRD-BP cDNA was a generous gift from Dr. Jeffrey Ross, University of Wisconsin. The mouse CRD-BP cDNA in this plasmid is flanked with the FLAG tag epitope at its N-terminus and a 6xHis-tag at its C-terminus. Recombinant CRD-BP was purified from *Escherichia coli* BL21(DE3) cells using a 1 mL bed volume of nickel-NTA (QIAGEN) column under denaturing conditions. Proteins eluted from the column at either pH 5.4 or 4.5 were subjected to a series of dialysis steps (3 hours at each step) pH 5/6 M urea, pH 5.5/4 M urea, pH 6/2 M urea, pH 6.7/1M urea and pH 7.4/0 M urea in a buffer containing 200 mM KCl, 1 mM EDTA, 10% (v/v) glycerol, 1 mM reduced glutathione, 0.1 mM oxidized glutathione, 0.01% (v/v) Triton X-100, 20 mM triethanolamine [13], and EDTA-free protease inhibitor tablets (Roche, Laval, Quebec). Following dialysis, the samples were centrifuged at 13,200 rpm for 30 minutes to remove any precipitated proteins. The purified protein solutions were then quantified using the Quick Start Bradford 1 x Dye Reagent (Bio-Rad, Mississauga, Ontario) and analyzed for purity using Coomasie blue-stained 12% SDS-PAGE.

### Generation of DNA templates used for *in-vitro* transcription

The previously constructed plasmid pUC19-CRD*myc-*1705-1886 was used to amplify the DNA template, which was used to synthesize internally radiolabeled 182 nts c-*myc* CRD RNA [33]. To make human β–globin RNA corresponding to nts 1–145, the plasmid SPĸβc was linearized with FokI and transcribed using SP6 Megascript kit (Life Technologies). The plasmid pCYPAC2 which contains the last CD44 exon, was a gift from Dr. Finn C. Nielsen (University of Copenhagen, Denmark), and was used as template for PCR amplification. The PCR primers used to amplify Fragment 4, 5, 8 and 9 DNA corresponding to the 3′untranslated region of CD44 mRNA are listed in Table 1. PCR amplified DNA templates were used directly for *in-vitro* transcription by T7 RNA polymerase. Oneμg of DNA template was incubated for 1 hour at 37°C in a 20-μl reaction containing 1 x transcription buffer (Promega, Madison, Wisconsin), 10 mM dithiothreitol, 1 unit RNasin (Promega), 0.5 mM ATP, 0.5 mM CTP, 0.5 mM GTP, 12.5 μM UTP, 1.5 units T7 RNA polymerase (Promega, Madison, WI), and 40 μCi [α-^32^P] UTP (3000 Ci/mmol). Following incubation, 3 units of RNase-free DNase I (Promega) were added and the reaction was further incubated for 10 minutes at 37°C. Upon addition of 10 μl Stopping dye (9 M urea, 0.01% bromophenol blue, 0.01% xylene cyanole FF, 0.01% phenol), the entire sample was electrophoresed on a 6% polyacrylamide/7 M urea gel and the band containing internally-radiolabeled RNA was excised and eluted with elution buffer (10 mM Tris-HCl pH 7.5, 0.1 M NaCl, 1 mM EDTA, 0.01% SDS) at 45°C for 6 hours. The purified, radiolabeled RNA was then phenol/chloroform extracted followed by ethanol precipitation. Specific activity of the RNA was then determined by scintillation counting.

### Electrophoretic mobility shift assay

The electrophoretic mobility shift assay (EMSA) binding buffer (5 mM Tris-Cl pH 7.4, 2.5 mM EDTA pH 8.0, 2 mM DTT, 5% glycerol, 0.1 mg/ml bovine serum albumin, 0.5 mg/mL yeast tRNA, 5 units RNasin) [24] was prepared on ice prior to each experiment. In order to facilitate RNA denaturation and renaturation, the [^32^P] RNA sample was heated to 50°C for 5 minutes and cooled to room temperature before adding the EMSA binding buffer and the appropriate amount of purified recombinant CRD-BP to a final volume of 20-μL. Reactions were incubated at 30°C for 10 minutes and transferred to ice for 5 minutes. The reaction was again incubated at 30°C for 10 minutes and transferred to ice for an additional 5 min. A total of 2 μL EMSA loading dye (250 mM Tris-Cl pH 7.4, 0.2% bromophenol blue, 0.2% xylene cyanol, 40% sucrose) was added to each reaction and 15 μL of the EMSA reaction was loaded onto an 8% native polyacrylamide gel and resolved at 25 mA for 90 minutes. Following electrophoresis, the gel was exposed overnight at –80°C and subjected to autoradiography using the Cyclone PhosphorImager and Optiquant Software.

EMSA saturation binding experiments were carried out as described above and the dissociation constant (K_d_) for the CRD-BP-CD44 RNA (nucleotides 2862–3055) interaction was determined using the Hill equation. The saturation binding data was analyzed by densitometry of the autoradiograph using the Cyclone Storage Phosphor-System and Optiquant software. For each reaction the total activity in each lane was determined; this involved summing the total activity in bound complexes with the total activity present in the unbound fraction. The percentage of bound RNA and the protein concentration (nM) were inserted into the Hill equation and the results were expressed graphically.

EMSA competition and inhibition assays involved the pre-incubation between competitor molecules (oligonucleotides, aminoglycosides or RNA) and CRD-BP for 10 minutes at 30°C. Following the pre-incubation, 40 nM radiolabeled probe was added to the reaction. This was followed by the standard EMSA protocol as described above. The molar excess concentrations of oligonucleotides over the radiolabeled RNA probe are shown in each figure.

### RNA isolation and quantitative real-time PCR

Total RNA was extracted from cells using the AMBION’s MirVana kit (Life Technologies) as according to the manufacturer’s instructions. CD44, β–actin, APE1, and c-*myc* mRNA levels were examined by quantitative real-time PCR (qPCR). The first strand cDNA synthesis was performed using iScript cDNA Synthesis kit (Bio-Rad, Mississauga, Ontario) on 1 μg of total RNA, and the qPCR was performed using iQ SYBR Green Supermix (Bio-Rad) on an iQ5 Multicolor Real-Time PCR Detection System (Bio-Rad). The PCR primers synthesized by IDT Inc. were: CD44 forward primer, 5′-CAT CAG TCA CAG ACC TGC CCA ATG C-3′, and CD44 reverse primer, 5′-ATG TAA CCT CCT GAA GTG CTG CTC C-3′; APE1 forward primer, 5′-TGG AAT GTG GAT GGG CTT CGA GCC-3′, and APE1 reverse primer, 5′-AAG GAG CTG ACC AGT ATT GAT GA-3′; c-*myc* forward primer, 5′-ACG AAA CTT TGC CCA TAG CA-3′, and c-*myc* reverse primer, 5′ GCA AGG AGA GCC TTT CAG AG-3′; β-actin forward primer, 5′-TTG CCG ACA GGA TGC AGA AGG A-3′, and β-actin reverse primer, 5′-AGG TGG ACA GCG AGG CCA GGA T-3′. The cycling protocol consisted of 95°C for 3 minutes and 40 cycles of denaturation at 95°C for 10 seconds, and annealing at 52°C for 30 seconds. To confirm amplification specificity, we performed a melting curve analysis at the end of each cycle. Each sample was amplified in triplicate, and the data were analyzed using iQ5 optical system software. Serial dilutions were carried out for each total RNA sample and reverse-transcribed under the above-mentioned conditions for each primer set to ensure amplification with efficiencies near 100%. C_T_ values for target genes (CD44, APE1, and c-*myc*) and reference gene (β-actin) were then used in the comparative C_T_ method or commonly known as the 2^−ΔΔCT^ method [34] to determine the expression level of a target gene in treated samples relative to the control DS Scrambled Negative-treated sample.

### Immuno-precipitation of the CRD-BP-RNA complex

HeLa cells transfected with pcDNA-FLAG or pcDNA-FLAG-CRD-BP in a 100 mm dish were lysed with 1 mL Total Cell Lysis (TCL) buffer (50 mM Tris-Cl pH 7.4, 150 mM NaCl, 1 mM EDTA, 0.1% Triton X-100) supplemented with 1 mM vanadyl ribonucleoside, 0.5 mM DTT, 0.05 units RNasin, and protease inhibitor tablet (Roche Diagnostics, Laval, Quebec). After 5 minutes on ice, lysed cells were aspirated using a 26 gauge needle five times to break the nuclei. After a 30-minute incubation on ice, the cell lysate was spun down at 14,000 rpm for 10 minutes. The collected supernatant was subjected to pre-clearing by incubating with 50 μL equilibrated-protein-G agarose beads (50% slurry) at 4°C for at least 1 hour. The resin was spun down at 3000xg for 1 minute and the lysate was collected. The pre-clearing step was repeated once after which the pre-cleared lysate was added to 5 μL anti-FLAG antibody (#F1804, Sigma-Aldrich, Oakville, Ontario) and mixed overnight at 4°C. Protein G-agarose beads were then added to the antibody-lysate mixture and mixed for 4 hours at 4°C to capture the anti-FLAG antibody. The agarose beads were then washed four times with TCL buffer followed by five washes with TCL buffer containing 1 M urea. Following the 4^th^ wash with TCL buffer containing urea, 50% of the agarose beads were collected and spun down at 3000xg for 1 minute followed by resuspension in 16 μL of ddH_2_O for Western blot analysis. The remaining 50% of the agarose beads were subjected to a final wash followed by resuspension in 100 μL TCL buffer containing 0.3 mg/mL proteinase K and 0.1% SDS and incubated at 50°C for 30 minutes. Following incubation, RNAs physically associated with the resin were extracted by the phenol-chloroform-isoamyl alcohol method and quantified using a Nanodrop Spectrometer (Wilmington, Delaware). One μg of RNA from each sample group was treated with DNase (DNA-free kit, Ambion) before cDNA synthesis and qPCR as described above.

The protocol for immuno-precipitating endogenous CRD-BP-RNA complex was the same as that described above for immuno-precipitation of FLAG-CRD-BP-RNA complex, except that protein A agarose beads and polyclonal IMP1 antibody (RN007P from MBL, Nagoya, Japan) were utilized.

### Western blot analysis

Protein samples were separated in a 12.5% polyacrylamide/SDS Lammeli gel system, transferred to a nitrocellulose membrane, and incubated against anti-FLAG (#200472-21, Stratagene, La Jolla, California), IMP1 polyclonal antibody (MBL, Nagoya, Japan), and β–actin (#A5441, Sigma-Aldrich) antibody. PageRuler Plus Prestained Protein Ladder (Thermo Fisher Scientific, Rockford, Illinois) was used to approximate the molecular weight of FLAG-CRD-BP, endogenous CRD-BP, and β–actin bands.

## Results

### Specific RNA sequences corresponding to the 3′ untranslated region of CD44 mRNA bind to CRD-BP

Using twelve *in-vitro* transcribed RNA fragments that cover the entire 3′UTR of the CD44 transcript, a previous study has identified five high affinity RNA binding sites for recombinant IMP1 [28]. The high affinity binding sites were detected in the 3′UTR at positions 2303–2627, 2862–3055, 3928–4236, 4567–4773 and 4749–5040 [28]. To better understand the interaction between the CD44 mRNA transcript and recombinant mouse CRD-BP and to eventually find molecules capable of breaking the CD44 RNA-CRD-BP interaction, RNA fragments were generated by *in-vitro* transcription corresponding to the highest affinity sites (Fragment 4: 2862–3055, and Fragment 8: 3928–4236) and to the lowest affinity binding sites (Fragment 5: 3565–3835, and Fragment 9: 4646–4910). The four ^32^P-internally labeled RNA fragments were assessed for binding to four concentrations of purified recombinant mouse CRD-BP ranging from 269 nM to 1885 nM. At the lowest concentration of CRD-BP, 14.1% and 11.5% of Fragment 4 and 8 were bound to the protein (Figure S1, lanes 2 and 17). In contrast, at the same concentration of CRD-BP only 4% and 7% of Fragment 5 and 9 RNA were bound respectively (Figure S1, lanes 7 and 12). This is in agreement with the previous finding that Fragment 4 and 8 have higher affinity than Fragments 5 and 9 for the human IMP1 [28]. Similarly, at the highest concentration (1885 nM) of CRD-BP, both Fragment 4 (83.6% bound) and 8 (81.4% bound) have higher affinity than Fragment 5 (58.9% bound) and 9 (63.8% bound) (Figure S1). In summary, our results are in agreement with the previous finding that Fragment 4 and 8 have higher affinity for CRD-BP. We also show that Fragment 5 (Figure S1, lanes 14 and 15) and 9 (Figure S1, lane 10) have affinity for CRD-BP only when higher concentrations of the protein were used.

### Characterizing the CD44 Fragment 4-CRD-BP Interaction

**Figure 1:**
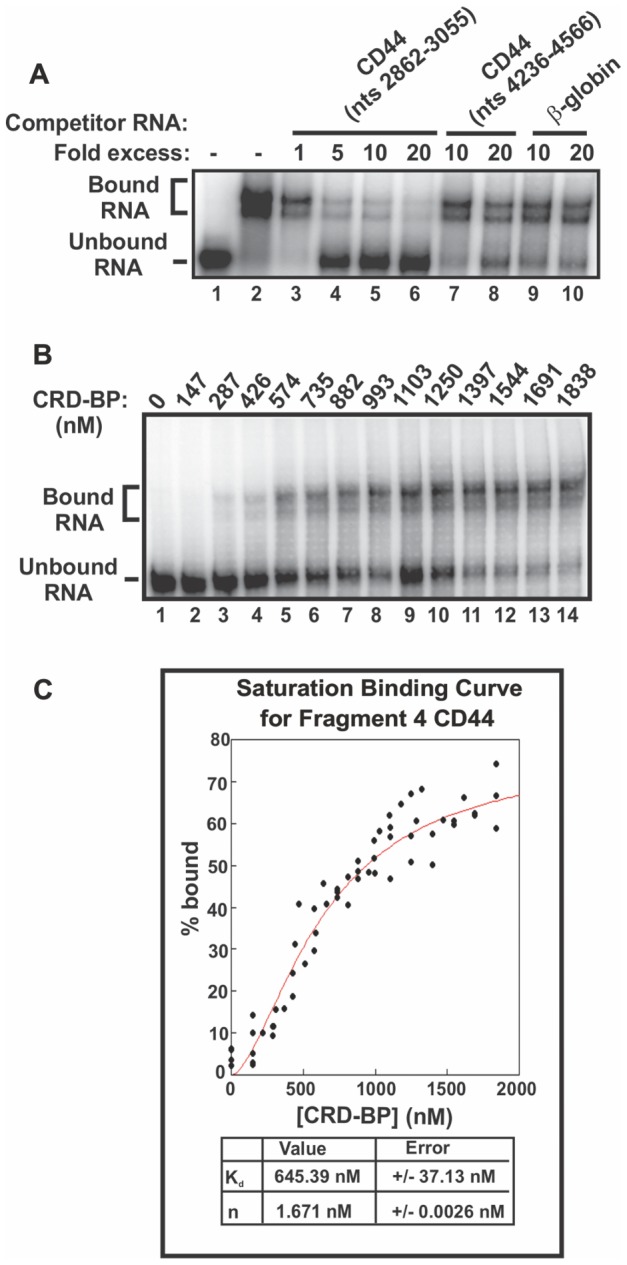
CRD-BP binding affinity for CD44 Fragment 4. (**A**) Purified His_6_-tagged CRD-BP (2000 nM) was incubated with [^32^P] CD44 Fragment 4 nts 2862–3055 (40 nM, 40,000 c.p.m) without (lane 2) or with molar excess of unlabeled competitor RNA CD44 Fragment 4 (lanes 3–6), CD44 RNA nts 4236–4566 (lanes 7 and 8), or β–globin RNA nts 1–145 (lanes 9 and 10). Lane 1 has no CRD-BP added. (**B**) EMSA showing increasing amounts of recombinant CRD-BP binding to [^32^P] CD44 Fragment 4. (**C**) A representative saturation binding curve is shown for CRD-BP binding to CD44 fragment 4. The K_d_ and Hill coefficient was averaged from replicate binding experiments using three different batches of recombinant CRD-BP.

We further characterized the interaction between CRD-BP and Fragment 4, which corresponds to position 2862-3055 of the CD44 transcript. We first performed EMSA competition assays to determine if the interaction between Fragment 4 and CRD-BP is specific. Figure 1A shows that unlabeled Fragment 4 dose-dependently competes with the ^32^P-labeled Fragment 4 (lanes 3–6) while up to a 20-fold excess of β–globin RNA (lanes 9 and 10) and CD44 RNA corresponding to position 4236–4566 (lanes 7 and 8) have no effect on ^32^P-labeled Fragment 4 binding to CRD-BP. This confirms that the interaction between Fragment 4 and CRD-BP is specific.

We performed saturation-binding experiments using ^32^P-labeled Fragment 4 to better understand the affinity of the interaction between CD44 RNA and CRD-BP. The saturation-binding experiments were done using three different batches of recombinant CRD-BP obtained from replicate purifications. A representative result for the binding of CRD-BP to CD44 ^32^P-labeled Fragment 4 is shown in Figure 1B. When the saturation-binding data from Figure 1B and two other experiments were fit to the Hill equation and expressed graphically, a sigmoidal curve was generated as shown in Figure 1C. The dissociation constant (K_d_) and Hill coefficient (n) were estimated to be 645 nM and 1.7 nM respectively. The Hill coefficient suggests that two molecules of CRD-BP may bind to one molecule of CD44 Fragment 4. Interestingly, similar binding characteristics (affinity and binding stoichiometry) of CRD-BP to a specific coding region of c-*myc* and *MDR*1 RNAs have been previously observed [24].

**Figure 2:**
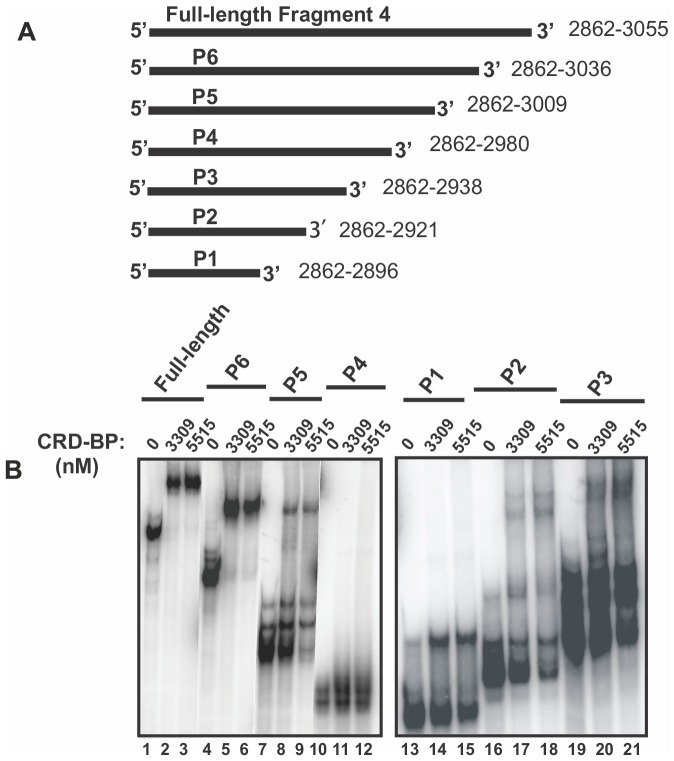
Assessing the ability of truncated CD44 Fragment 4 to bind CRD-BP. (**A**) A schematic diagram of the full-length CD44 Fragment 4 and its 3′ truncated RNAs, P1 to P6, used in EMSA to assess binding to CRD-BP. (**B**) EMSA demonstrating the ability of [^32^P] CD44 Fragment 4 (40 nM) and its 3′ truncated RNAs (P1 to P6) binding to recombinant CRD-BP at 3309 nM and 5515 nM. The relative sizes of the unbound and bound RNA for the full-length Fragment 4, P6, P5 and P4 on the non-denaturing polyacrylamide gel are shown (left panel). Similarly, the relative sizes of the unbound and bound RNA for P1, P2, and P3 are shown (right panel).

To determine the minimal size and sequence of Fragment 4 required for binding CRD-BP, we generated six 3′ end truncated Fragment 4 RNAs by *in-vitro* transcription (P1 to P6, Figure 2A) and then performed gel shift assays as before. The left panel in Figure 2B compares the ability of P6, P5, P4 and the full-length Fragment 4 to bind to two concentrations of CRD-BP (3309 and 5515 nM). The right panel in Figure 2B compares the ability of P1, P2 and P3 RNA to bind to CRD-BP. Each of the two panels in Figure 2B also compared the sizes of the unbound and bound ^32^P-labeled RNAs. The results in Figure 2B clearly show that the 175 nts P6 RNA that corresponds to position 2862–3036 was similarly effective as the full-length 194 nts Fragment 4 in binding to CRD-BP. Nearly all of the free labeled RNA was bound at a CRD-BP concentration of 3309 nM (lane 2 and 5). In contrast, the 148 nts P5 RNA exhibited weaker binding to CRD-BP, with only 15% of the RNA bound at both CRD-BP concentrations (lanes 8 and 9). The 119 nts P4 RNA exhibited the weakest binding to CRD-BP, with no bound RNA detected at both CRD-BP concentrations (lanes 11 and 12). Interestingly, the smaller 77 nts P3 RNA demonstrated binding to CRD-BP although it had a significantly reduced ability to bind CRD-BP as compared to the full-length Fragment 4, with only 10% bound to both concentrations of CRD-BP (lanes 20 and 21). The smaller 60 nts P2 RNA displayed a further significant reduction in binding to CRD-BP, with only 5% bound to both concentrations of CRD-BP (lanes 17 and 18) while the smallest 35 nts P1 RNA exhibited improved binding ability (25% bound) as compared to P2 (compare lanes 14 and 15 to lanes 17 and 18). In summary, we found that with the exception of P6 RNA, all other 3′ end truncated RNAs of Fragment 4 exhibited greatly reduced or abolished ability to bind CRD-BP. The binding profiles of P5 and P4 suggest that nts 2980–3009 are important for CRD-BP-binding. Similarly, comparing the binding profiles of P3 and P2 suggests that nts 2921–2938 are also important for binding CRD-BP.

### Oligonucleotides DD4, DD7, and DD10 effectively inhibit CRD-BP binding to CD44 RNA

**Figure 3:**
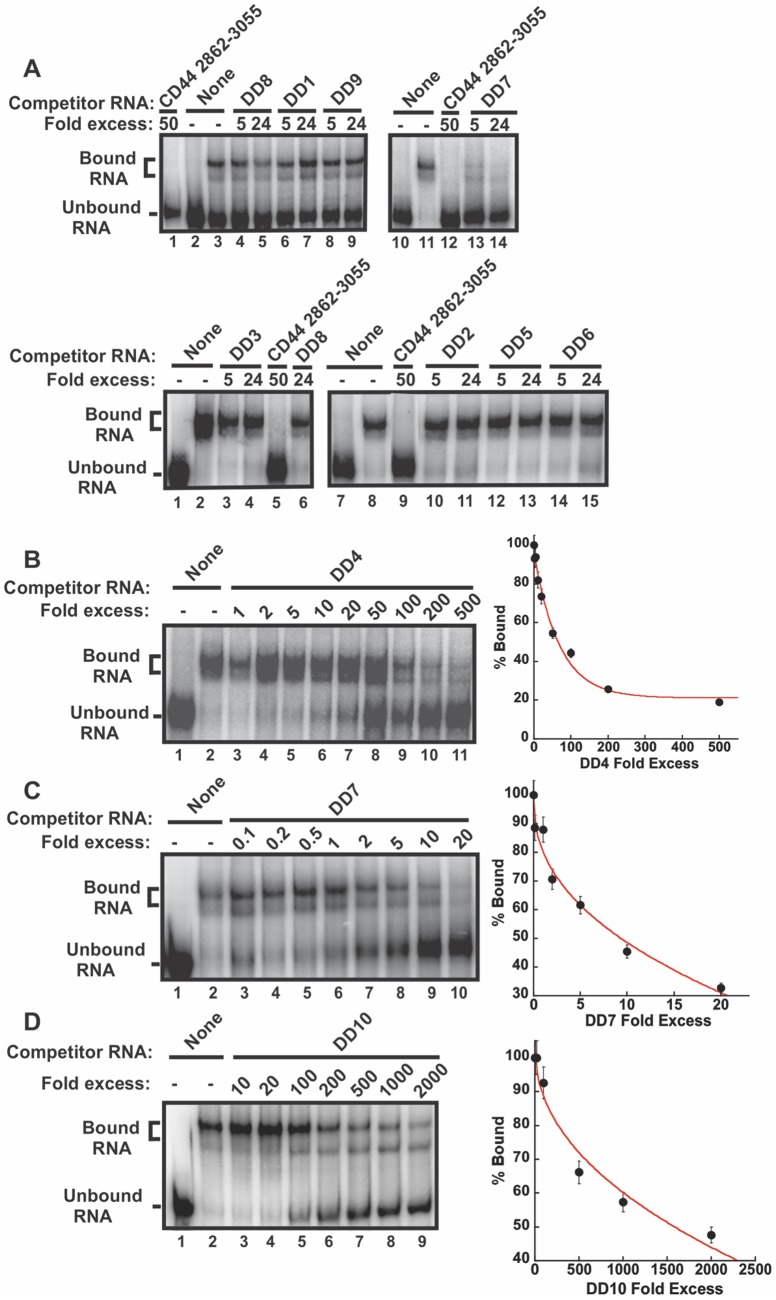
Inhibition of the CRD-BP-CD44 RNA interaction by antisense oligonucleotides. (**A**) Purified recombinant CRD-BP (2000 nM) was incubated with [^32^P] CD44 Fragment 4 in the presence of antisense oligonucleotides DD8 (lanes 4 and 5, upper panel), DD1 (lanes 6 and 7, upper panel), DD9 (lanes 8 and 9, upper panel), DD7 (lanes 13 and 14, upper panel), DD3 (lanes 3 and 4, lower panel), DD8 (lane 6, lower panel), DD2 (lanes 10 and 11, lower panel), DD5 (lanes 12 and 13, lower panel), or DD6 (lanes 14 and 15, lower panel). Samples in lanes 2 and 10 (labeled None, upper panel) and in lanes 1 and 7 (lower panel) had no CRD-BP added. In contrast, samples in lanes 3 and 11 (upper panel) and in lanes 2 and 8 (lower panel) had CRD-BP added. A fifty-fold excess of unlabeled CD44 Fragment 4 was used as a competitor positive control (lanes 1 and 12, upper panel; lanes 5 and 9, lower panel). Increasing amounts of oligonucleotides DD4 (**B**), DD7 (**C**), and DD10 (**D**) were incubated with [^32^P] CD44 Fragment 4 and purified recombinant CRD-BP. Lane 1 in Panel B, C, and D had no CRD-BP added. The average percentage of bound complex, taken from three separate experiments, is expressed on the graphs shown in the right panel of (**B**), (**C**), and (**D**).

**Figure 4:**
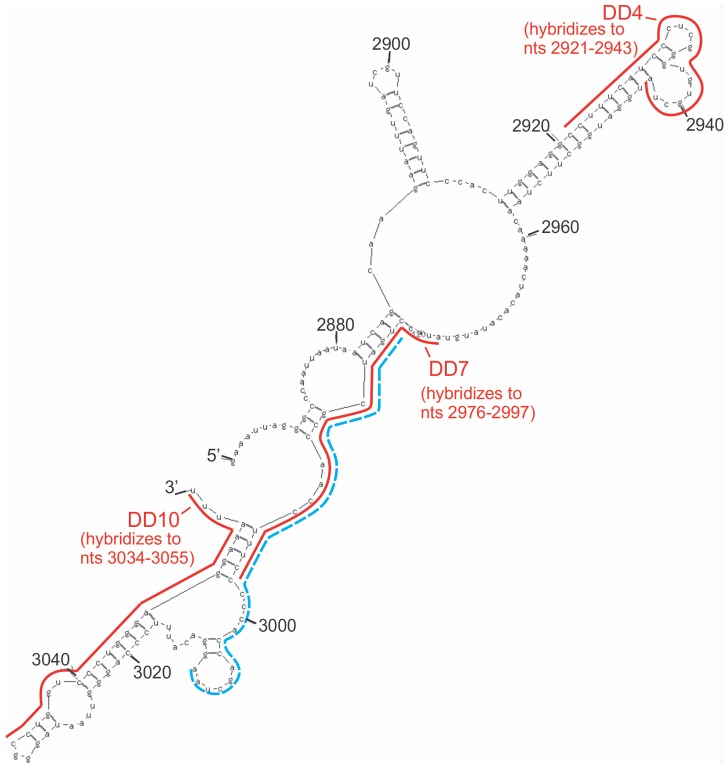
Predicted RNA secondary structure of Fragment 4 CD44 and target sites of antisense oligonucleotides. The predicted secondary structure of Fragment 4 (nts 2861–3055) CD44 was generated using the MFOLD program [53]. As shown, the solid lines indicate regions where DD4 (nts 2921–2943), DD7 (nts 2976–2997) and DD10 (nts 3034–3055) antisense oligonucleotides hybridize. The broken line from nts 2980–3009 indicates the missing region of RNA going from P5 to P4 truncated fragments (Figure 2).

We next investigated the possibility of using antisense oligonucleotides to inhibit the CRD-BP-CD44 RNA interaction. We designed ten antisense oligonucleotides each 22 nts in length, termed DD1 to DD10 (Table 1), which corresponded to the entire sequence of Fragment 4 CD44 RNA and assessed their ability to inhibit the binding of ^32^P-labeled Fragment 4 to CRD-BP. Figure 3A shows that DD1 (lanes 6 and 7, top panel), DD2 (lanes 10 and 11, bottom panel), DD3 (lanes 3 and 4, bottom panel), DD5 (lanes 12 and 13, bottom panel), DD6 (lanes 14 and 15, bottom panel), DD8 (lanes 4 and 5, top panel), and DD9 (lanes 8 and 9, top panel) were ineffective in inhibiting CRD-BP-CD44 Fragment 4 interaction even when used at up to 24-fold molar excess. In contrast, we found DD4, DD7, and DD10 to effectively inhibit the CRD-BP-CD44 Fragment 4 RNA interaction (Figure 3B to D), with DD7 being the most potent antisense oligonucleotide. At 10-fold excess, DD7 (Figure 3C) was able to inhibit 50% bound RNA, while DD4 (Figure 3B) and DD10 (Figure 3D) required 80- and 1500-fold excess to achieve similar level of inhibition. Upon examining the target sites of the antisense oligonucleotides, we found that the site (nts 2976–2997) targeted by the most effective competitor DD7 overlaps with the RNA region (nts 2980–3009) present in P5 RNA but missing in P4 RNA which resulted in no CRD-BP binding (Figure 4).

### Oligonucleotides DD4, DD7, and DD10 suppress the steady-state level of CD44 mRNA

**Figure 5:**
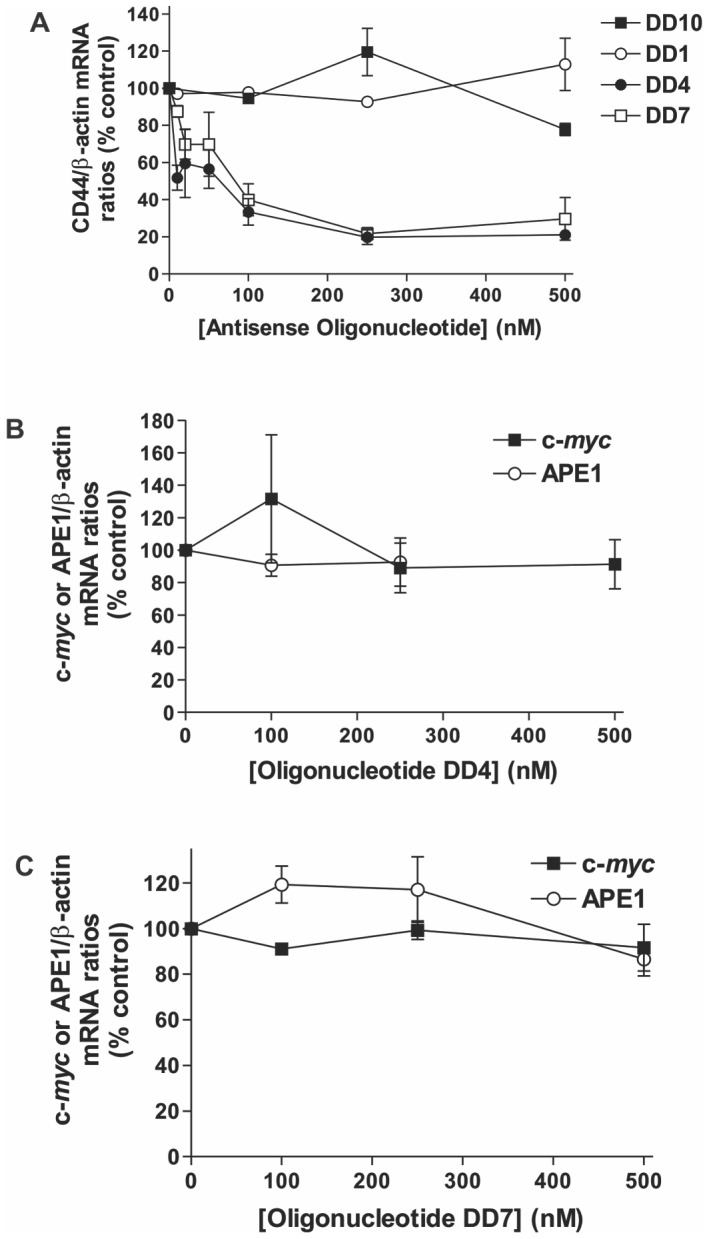
Effect of antisense oligonucleotides on CD44 mRNA expression in cells. HeLa cells were plated and transfected with various concentrations of 2′-*O*-methyl derivatives of DD1, DD4, DD7, or DD10 as described in the Materials and Methods. Total RNA extracted was subjected to quantitative real-time PCR for measurements of CD44, β-actin, c-*myc* and APE1 mRNAs. (**A**) Steady-state levels of CD44 mRNA was measured in cells using β-actin mRNA as a reference gene and as a function of increasing concentrations of DD1, 4, 7 and 10. Both DD4 and DD7 caused a reduction in CD44 mRNA levels in a concentration-dependent manner while DD1 and DD10 had no effect. Both DD4 and DD7 were tested for their effect on c-*myc* and APE1 mRNA levels as non-specific target RNAs as shown in (**B**) and (**C**). Data shown in all panels are expressed as a percentage of the control (non-transfected cells), and were averaged from three biological replicates with S.E. as error bars.

We next determined whether the most effective antisense oligonucleotides in inhibiting the CRD-BP-CD44 RNA interaction *in vitro*, namely DD4 and DD7, could influence the CD44 mRNA levels in cells. We used human cervical cancer HeLa cells because this cell line has been successfully used to demonstrate CRD-BP-mediated stabilization of CD44 mRNA [28]. Examination of the CRD-BP-CD44 mRNA interaction in multiple cell lines is an important avenue of future investigation but is beyond the scope of the present study. We transfected various concentrations of DD4, DD7, and DD10 into HeLa cells and then measure the levels of CD44 by quantitative real-time PCR using β–actin as the reference gene. DD1, being ineffective in breaking the CRD-BP-CD44 RNA interaction (Figure 3A), was used as a negative control. Figure 5A shows that transfection of either DD4 or DD7 suppressed CD44 mRNA levels even when used at a low concentration of 20 nM. At 100 nM of DD4 or DD7, there was a 60–70% reduction in CD44 mRNA levels and at the concentration of 250 nM, the reduction reached 80% for both oligonucleotides. In contrast, DD1 had no significant effect on CD44 mRNA expression up to 500 nM and DD10 only reduced the CD44 mRNA level by about 20% at 500 nM (Figure 5A). To determine if the suppressive effects by DD4 and DD7 on CD44 mRNA expression were specific, we also measured the mRNA levels of two other genes, c-*myc* and APE1. As shown in Figure 5B, DD4 did not affect either c-*myc* or APE1 mRNA expression. While DD7 had modest induction on APE1 mRNA level at 100 nM, no significant effect was observed at 300 and 500 nM (Figure 5C). At all concentration of DD7 tested, no effect was observed on c-*myc* mRNA level. In summary, we found specific suppression of CD44 mRNA expression by DD4 and DD7 but not by DD1 and DD10 antisense oligonucleotides.

### Oligonucleotide DD7 had no effect on the amount of CRD-BP-CD44 mRNA complex in cells

**Figure 6:**
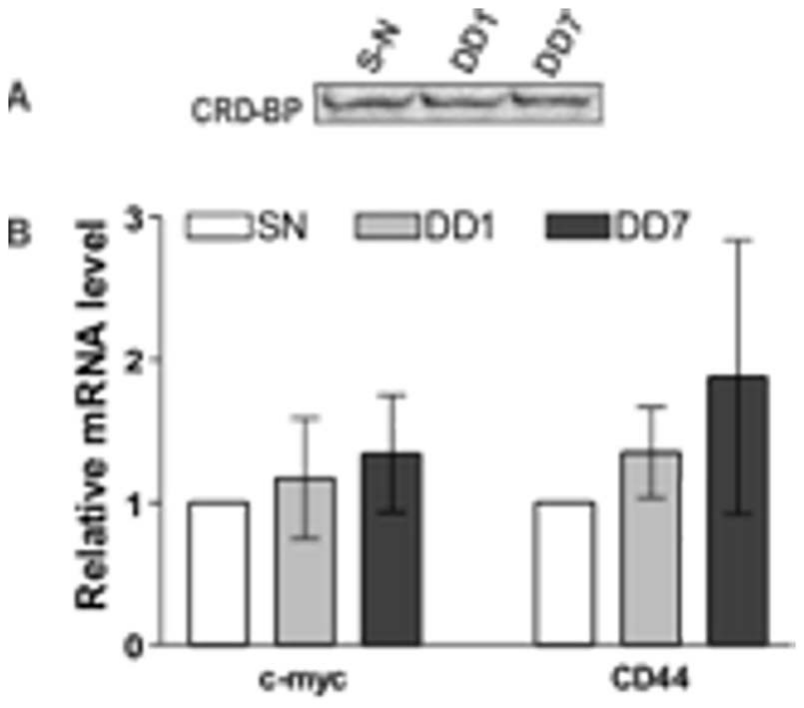
Effect of DD1 and DD7 antisense oligonucleotides on the CRD-BP-mRNA interaction in cells. HeLa cells were plated as described in the Materials and Methods. After a further 24-hour incubation, cells were transfected with 500 nM of 2′-*O*-methyl derivatives of DD1 or DD7, or with 500 nM of scrambled-negative (S-N) oligonucleotide, which served as a negative control. Immuno-precipitated endogenous CRD-BP was subjected to Western blot analysis using polyclonal IMP1 antibody (**A**). RNA physically associated with the immuno-precipitated endogenous CRD-BP was extracted as described in the Materials and Methods and subjected to quantitative real-time PCR to detect CD44 and c-*myc* mRNAs (**B**). The levels of CD44 and c-*myc* mRNA in DD1 and DD7-treated cells were expressed relative to that of S-N-treated cells which was presented as 1.0. Data shown were pooled from four separate biological replicates.

To determine whether the ability of oligonucleotide DD7 to specifically suppress the CD44 mRNA level is through a decrease in CRD-BP-CD44 mRNA complex formation, we performed immuno-precipitation experiments using polyclonal IMP1 antibody. The RNAs associated with the endogenous CRD-BP were extracted and subjected to quantitative real-time PCR for measurements of CD44 and c-*myc* mRNA levels. Figure 6A shows Western blot analysis of CRD-BP in cells transfected with 500 nM of DD7 oligonucleotide. Cells transfected with a scrambled-negative (S-N) oligonucleotide were used as a control. The DD1 oligonucleotide, which did not inhibit the CRD-BP-CD44 RNA interaction *in vitro* (Figure 3A) had no effect on the steady-state level of CD44 mRNA in cells (Figure 5A), and was used as a negative control. As shown in Figure 6A, cells transfected with 500 nM DD1, DD7 or S-N expressed equal amounts of CRD-BP. The relative amount of CD44 and c-*myc* mRNA extracted from an equal amount of immuno-precipitated CRD-BP from cells transfected with DD1, DD7 or S-N is summarized in Figure 6B. Our results from four separate experiments showed that there was no significant difference in the level of CD44 and c-*myc* mRNAs pulled out by CRD-BP in cells transfected with DD7 as compared to S-N. Hence, we concluded that the DD7 oligonucleotide has no effect on the overall abundance of CRD-BP-CD44 mRNA complex in cells.

### Assessing small molecule antibiotics for the ability to inhibit the CRD-BP-CD44 RNA interaction

**Figure 7:**
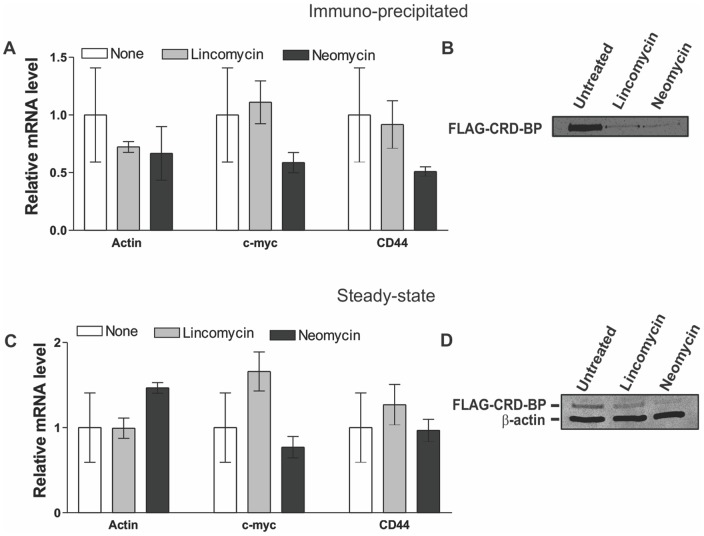
Effect of neomycin and lincomycin on the FLAG-CRD-BP-mRNA interaction in cells. HeLa cells were plated and transfected with pcDNA-FLAG-CRD-BP as described in Materials and Methods. Twenty-four hours after transfection, cells were treated with 750 μM of neomycin or lincomycin, or received no treatment. Immuno-precipitated FLAG-CRD-BP from cell lysate was subjected to RNA extraction and then quantitative real-time PCR (**A**) or Western blot analysis using anti-FLAG antibody as shown in (**B**). In a different set of experiments, HeLa cells transfected with pcDNA-FLAG-CRD-BP were directly subjected to total RNA extraction to assess steady-state levels of the c-*myc*, β-actin and CD44 mRNAs by quantitative real-time PCR (**C**). Total cell lysate was also analyzed to detect FLAG-CRD-BP protein expression in all groups, with β-actin used as a loading control (**D**). Data shown in (**A**) and (**C**) were pooled from three biological replicates.

Small molecules aminoglycoside antibiotics are known to interact with A-form nucleic acid molecules and interfere with RNA-protein interactions [35]–[38]. However, these compounds are classically utilized as anti-bacterial agents to target the RNA portion of the bacterial ribosome. To gain further insight into their ability to inhibit RNA-protein interactions, we investigated if common aminoglycosides can be used to inhibit the CRD-BP-CD44 RNA interaction. Using EMSA, we assessed the ability of nine different aminoglycoside antibiotics, over a concentration range from 0 to 1000 μM, for their ability to disrupt the interaction between CRD-BP and CD44 RNA Fragment 4. A summary of the half-maximal inhibitory concentration (IC_50_) of all nine small molecule antibiotics is shown in Table 2. The most potent inhibitor neomycin has an IC_50_ of 3.4 μM and paramomycin, the second most potent inhibitor has an IC_50_ of 58.8 μM. Interestingly, the IC_50_ value of neomycin for inhibition of the CRD-BP-CD44 RNA interaction is similar to its IC_50_ values in inhibiting other RNA-protein interactions such as the HIV Tat protein-TAR RNA interaction [37]. Kanamycin and streptomycin have IC_50_ values of 231.8 and 491 μM respectively. Puromycin, erythromycin, tobramycin, lincomycin and hygromycin were virtually ineffective in inhibiting CRD-BP-CD44 RNA interaction with IC_50_ values greater than 1000 μM. We next determined whether neomycin can affect the CRD-BP-CD44 mRNA interaction in cells. We first assessed whether HeLa cells can tolerate treatment with small molecules antibiotics, including neomycin, lincomycin and paromomycin, up to 1000 μM. Using light microscopy, we did not see any obvious morphological abnormalities exerted by the small molecule antibiotics even at 1000 μM (unpublished observation). We then treated cells over-expressing the FLAG-CRD-BP with 750 μM neomycin and assessed the level of mRNAs physically associated with FLAG-CRD-BP. In addition to non-treated cells being used as a negative control, we also treated cells with 750 μM of lincomycin, which has no inhibitory effect on CRD-BP binding to CD44 RNA *in-vitro* (Table 2). Figure 7A shows that treatment with either neomycin or lincomycin had no effect on the level of β-actin, c-*myc* or CD44 mRNA associated with CRD-BP. When we examined the amount of immuno-precipitated CRD-BP in each treatment group, we found that the level of FLAG-CRD-BP in cells treated with neomycin and lincomycin was significantly reduced when compared with non-treated cells (Figure 7B). To assess whether the reduced amount of immuno-precipitated FLAG-CRD-BP was due to reduced protein expression, we analyzed CRD-BP expression in total cell lysates from all groups. As shown in Figure 7D, indeed the expression of FLAG-CRD-BP was significantly decreased in neomycin- and lincomycin-treated cells as compared to the untreated cells. We also analyzed the mRNA levels in total RNA extracted from cells treated with 750 μM neomycin and lincomycin. As shown in Figure 7C, neither antibiotic had any effect on the steady-state level of CD44, c-*myc* and β-actin mRNAs. In summary, although neomycin effectively inhibited the CRD-BP-CD44 RNA interaction *in vitro*, its effect on the specific CRD-BP-CD44 mRNA interaction in cells was not apparent, likely masked by its global effects on protein-nucleic acid interaction within the cells.

**Table 2:** Half-maximal inhibitory concentration (IC_50_) of small molecule aminoglycoside antibiotics for inhibition of the CRD-BP-CD44 RNA interaction.

Aminoglycosides	IC_50_ (μM)
Neomycin	3.44
Paromomycin	58.8
Kanamycin B	231.8
Streptomycin	491
Puromycin	>1000
Erythromycin	>1000
Tobramycin	>1000
Lincomycin	>1000
Hygromycin B	>1000

The IC_50_ values were obtained from experiments conducted in a similar fashion to Figure 3.

## Discussion

It has become increasingly clear that CRD-BP plays an essential role in tumour progression. Although further *in-vivo* studies are required to clarify its mechanistic role, cumulative evidence points towards its ability to physically associate with target mRNAs as one mechanism for its oncogenic effect. To date, there have been only two studies that partially describe the use of molecules capable of blocking CRD-BP-RNA interactions [39], [40]. Further efforts toward finding inhibitory molecules are required because such compounds would have great therapeutic potential and can assist in elucidating the mechanism whereby CRD-BP binds to its wide selection of RNA targets. Herein, we describe the characterization of the CRD-BP-CD44 RNA interaction and evaluate a panel of antisense oligonucleotides and small molecule antibiotics for their ability to inhibit the CRD-BP-CD44 RNA interaction.

Using siRNA to knock down IMP1 in HeLa cells, Nielsen and co-workers have demonstrated the necessary role of IMP1 in cell adhesion, cytoplasmic spreading and invadopodia formation [28]. The CD44 mRNA transcript was substantially down regulated in IMP1-depleted cells and direct knockdown of CD44 mRNA exhibited phenotypes that resemble IMP1-depleted cells, suggesting that CD44 is a key player in IMP1-mediated cell adhesion and invadopodia formation [28]. Using EMSA, five high-affinity binding sites for recombinant IMP1 were identified in the 3′ UTR of CD44 mRNA [28]. Our study using a similar assay with recombinant mouse CRD-BP is consistent with previous findings (Figure S1). Both Fragment 4 and 8 RNAs spanning nts 2862–3055 and 3928–4236 respectively have high affinity for CRD-BP. However, both Fragment 5 and 9 RNAs spanning nts 3565–3835 and 4646–4910 have substantially lower affinity for CRD-BP (Figure S1). It is noteworthy however that at high concentrations, all fragments tested had affinity for CRD-BP suggesting that the 3′-UTR may have multiple high and low affinity binding sites and more than one of these sites likely contribute to the binding of the entire mRNA transcript.

The binding affinity of CRD-BP was strongest towards the 194 nts CD44 Fragment 4 RNA (K_d_ =  645 nM), with an affinity similar to other known binding partners such as c-*myc* and *MDR*1 RNAs [24], with K_d_ at around 600 nM. It is also worth noting that a second binding complex could be detected in all EMSAs performed with all CD44 RNA fragments tested. Interestingly, c-*myc* and *MDR*1 mRNAs all displayed a similar binding pattern in EMSAs [24]. Furthermore, we found that the Fragment 4-CRD-BP interaction had a Hill coefficient of n = 1.7, indicating positive cooperativity and further suggesting that perhaps two molecules of CRD-BP are binding to a single CD44 RNA molecule. In support of this hypothesis is a previous report where it was found that two molecules of IMP1 binds to *Igf-II* and *H19* RNA by a sequential, cooperative mechanism [41].

In further characterizing the CRD-BP-Fragment 4 CD44 RNA interaction, we found that, in general, smaller RNA fragments have a reduced affinity for CRD-BP. This has also been observed for the CRD-BP-c-*myc* RNA interaction [2]. We propose that the multi-RNA binding domain architecture of CRD-BP brings about the potential for simultaneous interaction of a single protein molecule with distinct RNA binding sites that are separated by large stretches of sequence. Corroborating this notion is the finding that the ZBP1 KH3-4 di-domain crystal structure adopts an intramolecular anti-parallel pseudodimer that induces a 180° looping of its bound RNA binding partner, necessitating a large RNA ligand for binding to both domains in tandem [5].

The largest reduction in CRD-BP binding was observed when truncating the CD44 RNA from fragment P5 (nts 2862–3009) to P4 (nts 2862–2980) (Figure 2), suggesting that nts 2980–3009 are important for binding to CRD-BP. In addition, our results show that the 35 nts CD44 RNA spanning nts 2862–2896 has affinity for CRD-BP although at a vastly reduced level compared to the full-length fragment 4 or P6 RNA fragment (Figure 2). Such information is valuable for the development of high-throughput assays to screen for inhibitors of the CRD-BP-CD44 RNA interaction, which require the use of small fluorescent RNA molecules such as in fluorescence anisotropy assays [40], [42].

Based on analogy to ZBP-1, the CRD-BP C-terminal KH domains are implicated in RNA binding rather than the N-terminal RNA recognition motifs [5]. It is well documented that KH domains bind only to regions of single stranded RNA [43]. Therefore, we reasoned that inhibition of RNA-CRD-BP complex formation via hybridization of an antisense oligonucleotide to the binding site should effectively inhibit the interaction and provide an excellent approach to attain maximal target specificity. We chose 22 nts oligonucleotides because this has been identified as the optimal length for both binding affinity and target specificity while still maintaining high cellular uptake [44].

Using the *in-vitro* gel shift assay, we tested ten antisense oligonucleotides, which collectively cover the entire CD44 Fragment 4, for the ability to inhibit CRD-BP binding to ^32^P-labeled CD44 Fragment 4. Only three of these antisense oligonucleotides, namely DD4, DD7 and DD10, were effective as competitors to occlude CRD-BP binding (Figure 3). DD10 was the least effective, requiring over a thousand-fold excess to compete for binding (Figure 3D). Interestingly, the sites targeted by the most effective competitor DD7 (nts 2976–2997) overlap with the RNA regions of Fragment 4 that are required for binding to CRD-BP (Figure 4). This further corroborates the finding that DD7 is the best competitor against CRD-BP. We then transfected the 2′-*O*-methyl derivatives of DD4, DD7 and DD10 into cells to assess their effectiveness in inhibiting CD44 mRNA expression. We also included a 2′-O-methyl derivative of DD1 for use as a negative control. Our results showed that both DD4 and DD7 were potent and specific inhibitors of CD44 mRNA expression with 80% suppression at 250 nM, while both DD1 and DD10 had no effect on CD44 mRNA levels (Figure 5). Thus, the effectiveness of the antisense oligonucleotide molecules in inhibiting the CRD-BP-CD44 RNA interaction *in-vitro* directly correlates with their ability to decrease CD44 mRNA levels in cells. Furthermore, these results further support the role of CRD-BP in directly stabilizing the CD44 mRNA transcript in cells [28].

Using immuno-precipitation coupled with the reverse transcription qPCR method we provide the first direct evidence that CRD-BP and CD44 mRNA do in-fact form a complex in cells. Intriguingly, we find that both DD1 and DD7 have no significant effect on the relative amount of endogenous CRD-BP-CD44 mRNA (Figure 6B) complexes formed in cells, despite the fact that DD4 and DD7 clearly destabilize CD44 mRNA transcript levels (Figure 5). As shown using *in-vitro* EMSA assays, there are multiple CRD-BP binding sites scattered throughout the CD44 mRNA 3′UTR region. Taken together, this data suggests that the interaction between CD44 mRNA and CRD-BP may have more complex global stoichiometry than previously appreciated. In this light, it is not surprising that antisense oligonucleotide mediated disruption of a single CRD-BP binding site does not show a drastic detectable effect on the relative *in-vivo* abundance of CRD-BP-CD44 mRNA complex. This leads us to propose a tentative model whereby addition of DD4 and DD7 disrupt the local association of CRD-BP, however do not drastically alter the global binding of CRD-BP to the entire 3′UTR region. Interruption of CRD-BP binding at these “hotspots” may open up the transcript for nuclease- or microRNA-mediated degradation of CD44 mRNA. We believe that the observed CD44 mRNA transcript destabilization is not due to the RNase H pathway as the 2′-O-methyl derivatives utilized in this study are highly resistant to RNase H-mediated degradation [45]. It is possible that a similar multi-binding site interaction model may be operative for other CRD-BP target transcripts. Indeed, CRD-BP is known to physically associate with many other RNA-binding proteins and mRNAs in the cytoplasmic ribonucleoprotein granules [46], [47]. Whatever the specific mechanism are, our results reveal that DD4 and DD7 specifically destabilize the CD44 mRNA transcript. Therefore, antisense oligonucleotides may be a valuable approach to the *in-vivo* destabilization of mRNA transcripts via targeting RNA-binding protein interaction sites.

We also evaluated a panel of aminoglycoside antibiotics including some that have previously been used to break protein-RNA interactions [35]–[38] for their ability to block the CRD-BP-CD44 RNA (2862–3055) interaction. Out of the nine aminoglycosides assessed, four effectively inhibited the CRD-BP-CD44 RNA Fragment 4 interaction *in vitro* (Table 2). Neomycin was the most potent with an IC_50_ value of 3.4 μM, which is similar to its known IC_50_ values in inhibiting other protein-RNA interactions [37], [38]. Our results point out that assessing the effect of aminoglycoside antibiotics on cell phenotype can be very difficult to interpret. This is mainly because aminoglycoside antibiotics are positively charged and likely have a myriad of other unidentified nucleic acid targets, resulting in inhibition of protein synthesis amongst various other cellular affects. Indeed, aminoglycosides can bind to the A-site of the 30S ribosomal subunit to interfere with codon recognition and translocation [48], [49]. The observed gene expression profile of neomycin- and lincomycin-treated cells (Figure 7) supports this hypothesis. The inability to destabilize the CD44 mRNA-CRD-BP complex upon treatment with neomycin or lincomycin (Figure 7A) suggests that these antibiotics lack specificity for the CRD-BP-RNA interaction and likely target other more abundant bio-molecules in cells. Compounds rich in positive charge, such as the aminoglycosides, are often pulled out from nucleic acid-based screening assays and are likely to have a non-specific activity when tested in cells. Neomycin is known for its avidity to A-form nucleic acids such as RNA, triplex DNA and has also been shown to induce DNA:RNA hybrid triplex formation [50]. Several intercalator-neomycin fusions have been designed, which displays greater affinity and specificity for binding to triplex DNA [50]–[52]. As exemplified in the present study, neomycin displays vast potential in nucleic acid binding ability, however, the challenge for the future will be the functionalization of the neomycin core scaffold to confer sequence specificity in RNA binding. It is also important to point out that aminoglycosides such as neomycin are known to have side effects on the kidney, vestibular and ear [54], [55]. Hence, any future exploration on the use of aminoglycosides has to take such limitation into consideration.

In conclusion, we have characterized the interaction between CRD-BP and CD44 RNA nts 2862–3055 and mapped the CD44 RNA interaction region down to 35 nts in size, which still has affinity for CRD-BP. Furthermore, we reveal three antisense oligonucleotides that can block the CRD-BP-CD44 RNA interaction *in-vitro* and are effective and specific in regulating CD44 mRNA levels in cells, suggesting that *in vitro* gel shift assays are a valuable tool for assessing the ability of nucleic acid-based molecules to inhibit the CRD-BP-RNA interactions. Our results also exemplify that caution must be used when considering the use of aminoglycosides to inhibit RNA-protein interactions because this group of molecules has a high propensity to non-specifically interact with a wide range of nucleic acid targets and thus alter multiple biological processes in cells. Taken together, this study represents a valuable step toward selectively regulating oncogenic gene expression by targeting CRD-BP-RNA interaction sites using antisense oligonucleotides.

## Supporting Information

Figure S1 **Binding of CRD-BP to the 3**′**UTR of CD44 mRNA.** EMSAs were performed (upper panel) with four different [^32^P] CD44 RNA fragments (40 nM), in the absence (lanes 1, 6, 11, 16) or presence of various concentrations of recombinant CRD-BP (lanes 2-5, 7-10, 12-15, and 17-20). The unbound and bound radiolabeled RNAs are indicated. The lower panel shows the positions of the individual RNA fragments (Fragments 4, 5, 8, and 9) corresponding to the 3′UTR of CD44 mRNA (NCBI accession number: NM_000610).
(TIF)
