# Supplementary figures and images for: Assessing Specific Oligonucleotides and Small Molecule Antibiotics for the Ability to Inhibit the CRD-BP-CD44 RNA Interaction
Source: PLoS One. 2014 Mar 12;9(3):e91585. doi: 10.1371/journal.pone.0091585 (PMC3951440; doi:10.1371/journal.pone.0091585)

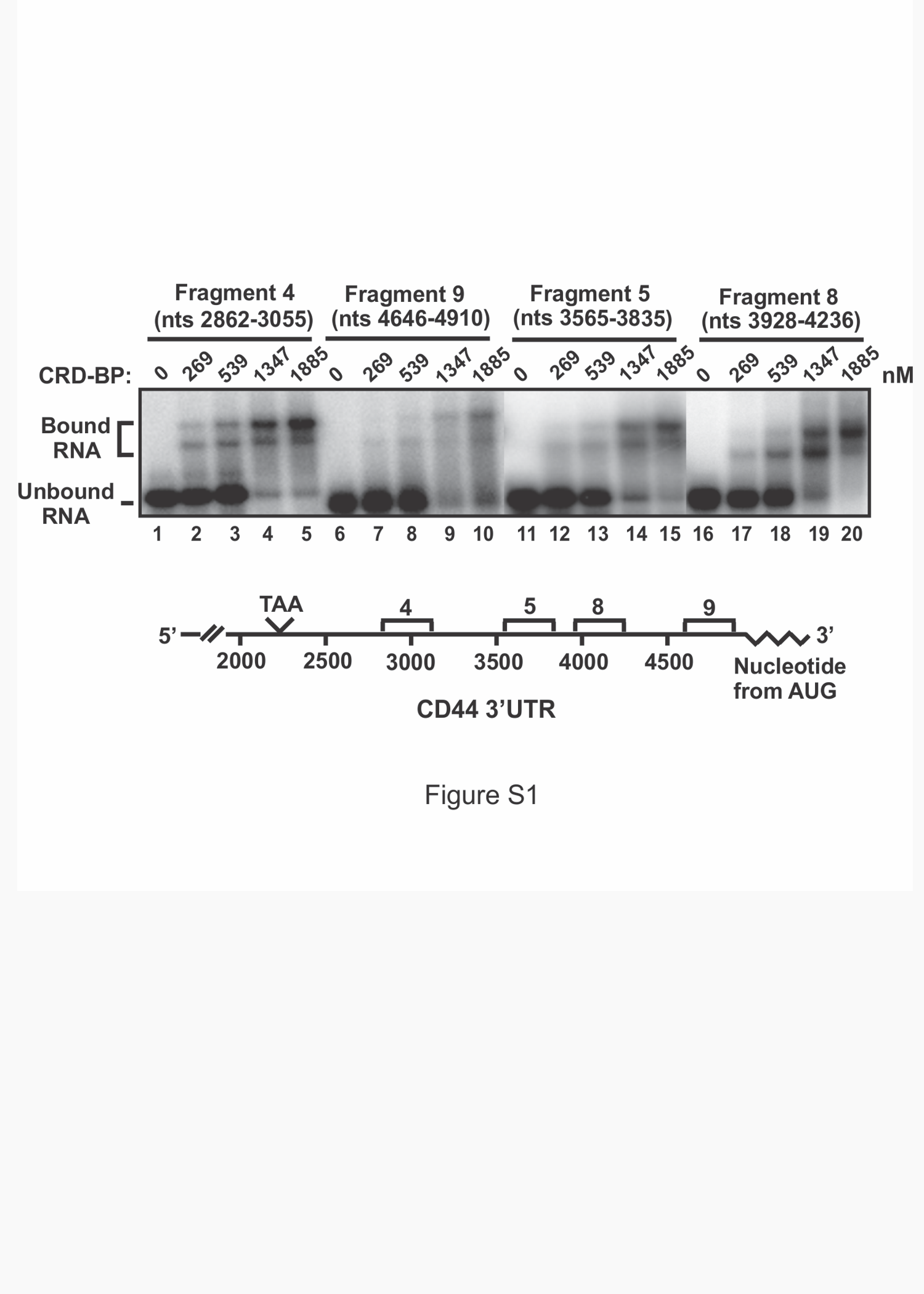

Supplement: Figure S1 — Binding of CRD-BP to the 3′UTR of CD44 mRNA. EMSAs were performed (upper panel) with four different [32P] CD44 RNA fragments (40 nM), in the absence (lanes 1, 6, 11, 16) or presence of various concentrations of recombinant CRD-BP (lanes 2-5, 7-10, 12-15, and 17-20). The unbound and bound radiolabeled RNAs are indicated. The lower panel shows the positions of the individual RNA fragments (Fragments 4, 5, 8, and 9) corresponding to the 3′UTR of CD44 mRNA (NCBI accession number: NM_000610). (TIF) [file pone.0091585.s001.tif]
